# Organizing organelles: bacterial strategies for localizing intracellular compartments

**DOI:** 10.1016/j.mib.2025.102614

**Published:** 2025-06-03

**Authors:** Yein Ra, Arash Komeili

**Affiliations:** Department of Plant and Microbial Biology, University of California, Berkeley, United States

## Abstract

Bacteria contain multiple subcellular compartments that enable a variety of biochemical activities and behaviors. In many cases, the organization of these organelles is not random and is directly linked to their function. In the last decade, mechanistic studies have uncovered the machinery responsible for organelle positioning in some bacterial systems. Here, we review several such positioning systems with an emphasis on two arrangement patterns and the molecular mechanisms used to achieve them. We start with carboxysomes as an illustration of how a ParA/MinD ATPase system is used to equally distribute organelles in a cell. We follow with an example of an actin-like cytoskeletal system that links lipid-bounded magnetosome organelles into a continuous chain. We finally explore emerging models of bacterial organelle positioning and conclude with an outlook on the future opportunities in the study of bacterial organelle cell biology.

## Introduction

Having organelles is no longer considered a strictly eukaryotic trait; we now know that a vast repertoire of organelles exists in bacteria [1]. These subcellular compartments, spanning from lipid-bounded ferrosomes to nanoscale encapsulins, are characterized by a distinct boundary with a specific internal composition that often enables the execution of otherwise inefficient or toxic biochemical reactions [2,3]. Functions of organelles include concentration of substrates with their enzymes, containment of toxic metabolites, and storage of energy-rich compounds [1]. Careful observations of organelle distributions reveal that many are not randomly positioned within the cell. Indeed, bacterial species have evolved dedicated positioning systems to localize organelles. One common motivation is to ensure the proper segregation of organelles during cell division, which is typically achieved by equally distributing organelles along the cell length [4,5]. Other times, an organelle’s position and organization are directly linked to its cellular function [6].

This review aims to summarize different strategies and the underlying molecular mechanisms of organelle positioning in bacteria. We first detail the current knowledge on the two best-studied examples of bacterial organelle positioning: the ParA/MinD family ATPase-based system of carboxysome organization and the cytoskeleton-based system of magnetosome chain formation. We next describe two emerging models of bacterial organelle positioning: the polyhydroxybutyrate (PHB) granule and the polyphosphate granule. We conclude with a generalized model of bacterial organelle organization and an outlook on future research endeavors in this area.

### Carboxysomes — spacing through McdA gradients

Organisms that fix carbon through the Calvin–Benson–Bassham cycle face an issue with ribulose-1,5-bisphosphate carboxylase/oxygenase (RuBisCO): the carbon-fixing enzyme also reacts with oxygen, leading to unproductive photorespiration. Cyanobacteria and other carbon-fixing proteobacteria have solved this problem by concentrating carbon dioxide in the same compartment that houses RuBisCO. This bacterial organelle — the carboxysome — has a proteinaceous shell and is the best studied bacterial microcompartment (BMC) [7].

**Figure 1:**
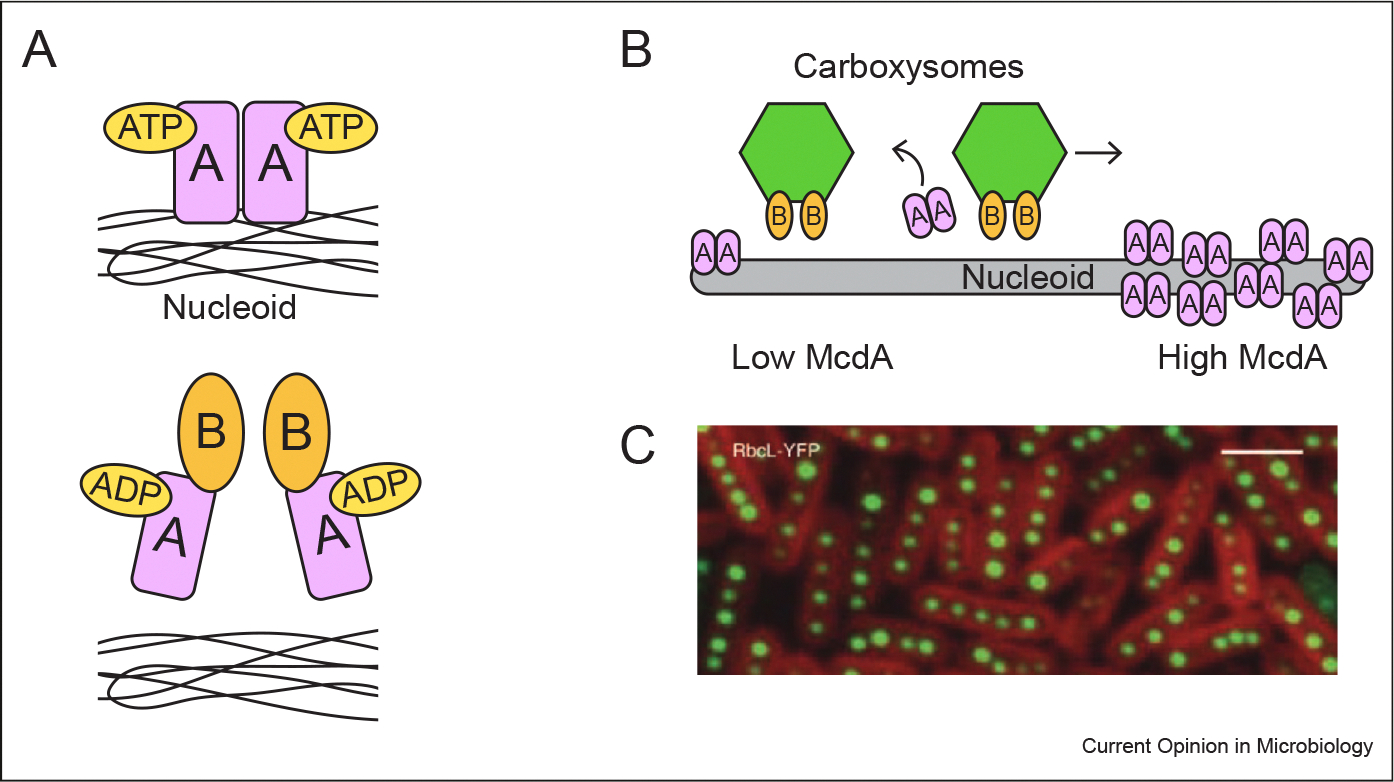
Carboxysomes are equally distributed along the nucleoid by McdA and McdB. **(a)** McdA dimerizes and binds to DNA nonspecifially when bound to ATP. McdB triggers the ATPase activity of McdA, which causes McdA to dissociate from DNA [8]. **(b)** McdB-bound carboxysomes create zones of depleted McdA while migrating toward areas of greater McdA concentration [8]. Note that oligomerization of McdB in some species is not depicted [9]. **(c)** Carboxysomes labeled by RbcL-YFP are equally spaced as a result of the McdAB system [4,8]. **(c)** Reprinted with permission from ref. [4].

Carboxysome positioning has been primarily studied in β-cyanobacterium *Synechococcus elongatus* PCC 7942 and chemoautotrophic proteobacterium *Halothiobacillus neapolitanus* [8,9]. In both organisms, carboxysomes are linearly distributed uniformly along the cell length by the action of two interacting proteins, McdA and McdB [4,8]. The importance of the McdA and McdB can be clearly seen via genetic analyses since deletion of *mcdA* and/or *mcdB* results in mislocalized and aggregated carboxysomes [4,8]. A series of elegant biochemical experiments shows that McdA and McdB work similarly to the well-described ParAB DNA segregation system [8,10]. McdA is a ParA-type ATPase [4,8]. When ATP-bound, it dimerizes and nonspecifically binds to the nucleoid [8,11,12]. McdB localizes to carboxysomes and triggers the ATPase activity of McdA [8]. This causes McdA to be released from DNA, creating zones of differing McdA concentrations along the nucleoid. Because McdB-bound carboxysomes migrate toward higher concentrations of McdA while also clearing nearby DNA-bound McdA, carboxysomes in close proximity are more likely to move away from one another. The overall result of the McdAB system is uniformly distributed carboxysomes along the nucleoid (Figure 1) [8].

Deeper investigation of the molecular workings of McdB has revealed a connection between carboxysome positioning and phase separation (PS), a process in which a molecule solution is separated into a dilute and concentrated phase [13]. Sequence analysis of over 500 predicted McdBs show that the protein varies widely in both sequence and length [9,14]. The few features shared among all McdB homologs, such as an intrinsic disordered domain, indicate that the ability to perform PS is conserved [9,14]. In *S. elongatus*, McdB PS is pH-dependent [14,15] and partially regulated by its positively charged N-terminal intrinsic disordered region [15]. Replacing the positively charged residues with glutamine drastically reduces McdB condensate formation and its ability to position carboxysomes [15]. The positively charged residues might also be required for interactions with McdA, therefore whether the mispositioning of carboxysomes is due to lack of PS or loss of McdA interaction remains unresolved. Another conserved feature across all McdB proteins is an invariant tryptophan at the C-terminal end. The tryptophan is necessary for McdB localization to carboxysomes and also affects its ability to form condensates [16]. Replacing the tryptophan with other aromatic residues partially restores McdB localization and PS ability to varying degrees. Greater condensate formation *in vitro* is correlated with higher frequency of McdB carboxysome localization, providing further evidence that PS plays a role in carboxysome targeting [16].

The conservation of *mcdAB* highlights the significance of correct carboxysome positioning [8,9,14]. Indeed, the equidistant distribution ensures equal division of carboxysomes between the two daughter cells after cell division [4]. Cells that inherit carboxysomes have shorter doubling times and greater carbon fixation rates compared to cells that synthesize all carboxysomes *de novo* [4]. Furthermore, *S. elongatus* strains with mispositioned carboxysomes have increased occurrence of elongated cells, asymmetrical cell division, slower growth rates, and higher RuBisCO levels when grown with high CO_2_ [17]. Given the importance of correct carboxysome positioning, it is unsurprising that *mcdAB* is present in the majority of genomes of carboxysome-producing organisms analyzed [9,14]. An unexpected exception is α-cyanobacteria, for which no *mcdAB* system could be identified [14]. Whether α-cyanobacteria have a dedicated carboxysome positioning system is still an open question.

Though most *mcdAB* operons are encoded near carboxysome genes, occasionally *mcdAB*-like genes are detected near operons of other BMCs, such as ethanolamine utilization microcompartments or 1,2-propanediol utilization microcompartments (PDU) [9]. This suggests that McdAB-like proteins position BMCs other than carboxysomes in selected organisms. It also appears that McdAB-like proteins are not the only BMC positioning system. For example, the minor shell protein PduK in *Salmonella enterica* serovar Typhimurium prevents aggregation of PDU at the cell poles [18]. More directed studies on the spatial organization of non-carboxysome BMCs will undoubtedly uncover novel organelle positioning strategies.

### Magnetosomes — linking through cytoskeletal filaments

Many microbes face the challenge of navigating toward desired nutrients or stimuli. To improve the efficiency of this process, magnetotactic bacteria (MTB) have evolved to align to and navigate along the geomagnetic field using magnetosomes, membrane-bounded compartments in which a magnetic crystal is biomineralized [19]. This behavior is thought to simplify the organism’s search for the oxic-anoxic transition zone of the water column, a process termed magnetoaerotaxis. A prerequisite to magnetoaerotaxis is the formation of a linear chain of magnetosomes, allowing individual magnetic crystals to collectively act as a single compass needle to orient the cell [19]. MTB strains with aggregated or dispersed magnetosomes have severely impaired magnetoaerotaxis [6,20], exemplifying how an organelle’s position can directly affect its function.

**Figure 2:**
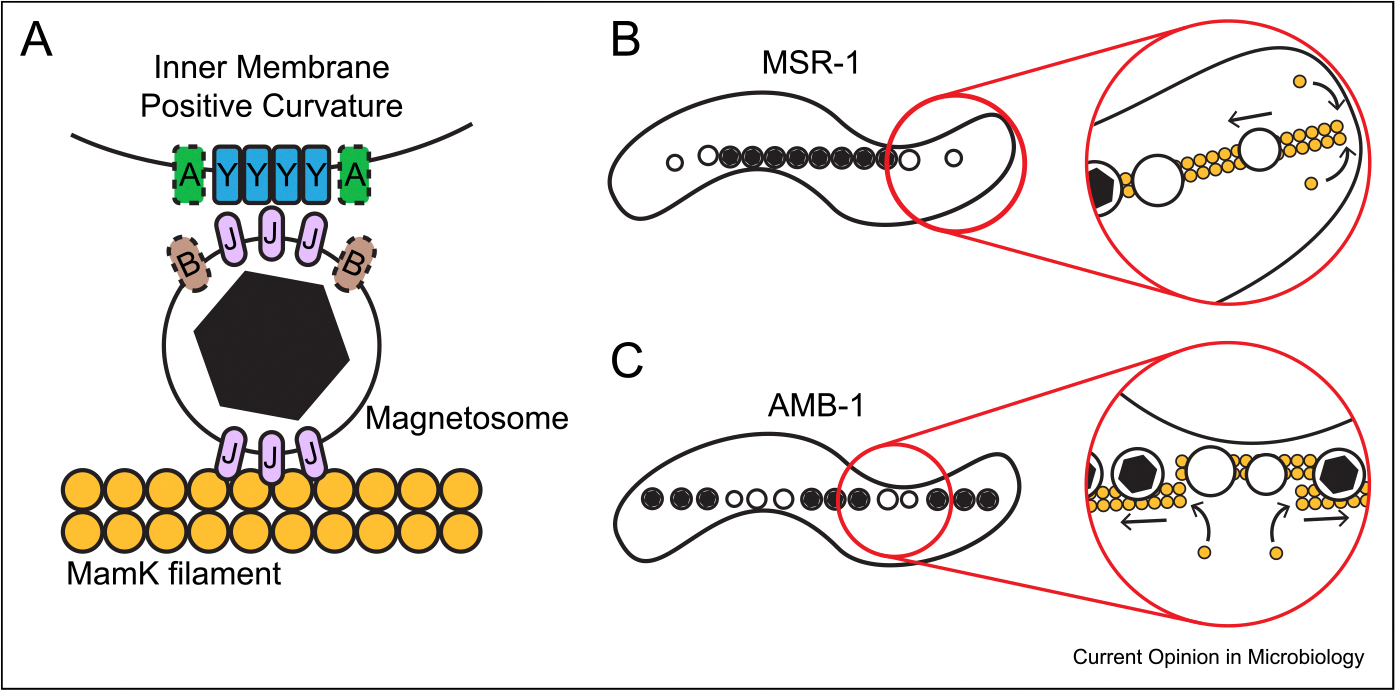
Magnetosomes are positioned by a cytoskeletal system. **(a)** The lipid-bound magnetosome in MSR-1 and AMB-1 associates with the MamK filament and positive curvature-localized MamY through MamJ [6,22]. In AMB-1, McaA is found at the positive curvature, and McaB localizes to crystal-containing magnetosomes [29]. **(b)** In MSR-1, magnetosomes without magnetic crystals are at the chain periphery. MamK polymerization at the cell poles pushes magnetosomes toward the midcell, maintaining a continuous, linear chain [25]. **(c)** In AMB-1, McaA and McaB organize the magnetosome chain such that there is an alternating pattern of older magnetosomes (with magnetic crystals) and newer magnetosomes (without magnetic crystals). This pattern is speculated to arise from MamK pushing older magnetosomes into subchains, opening up space for new magnetosomes to form in the gaps [29].

Magnetosome chain assembly and positioning have been best studied in two model MTB, *Magnetospirillum gryphiswaldense* MSR-1 and *Magnetospirillum magneticum* AMB-1 (referred to as MSR-1 and AMB-1, respectively). Both species employ a similar cohort of proteins to assemble their magnetosome chain (Figure 2) [19]. The system’s three key components–MamK, MamJ, and MamY–work in concert to form a cohesive magnetosome chain centered along the positive curvature of the inner cell membrane [6,21,22].

At the core of building a magnetosome chain is MamK, which forms a distinct branch of the bacterial actin-like family of proteins [21]. MamK polymerizes into a collection of parallel filaments, spanning from cell pole to pole, on which magnetosomes attach [21,23]. Similar to other actins, MamK monomers polymerize into double-stranded filaments when bound to ATP [24]. While incorporated in the filament, MamK hydrolyzes its ATP to ADP, which favors monomer disassociation [24]. The ATP-dependent polymerization and depolymerization give MamK its dynamic behavior. In MSR-1, MamK polymerization occurs at the cell poles, which pushes the connected magnetosomes toward the midcell to establish a single, continuous chain (Figure 2b) [25]. The centered magnetosome chain that becomes polarly localized due to cell division also relies on MamK to migrate to the new midcell [25]. Removing MamK’s ATPase activity or deleting *mamK* causes large gaps between magnetosomes, reduction in the cell’s ability to align with a magnetic field, and unequal magnetosome segregation during cell division [21,23,25–27]. Thus, MamK governs the spacing between magnetosomes in the cell.

MamK’s function in this system requires it to interact with magnetosomes. This role is filled by the acidic protein MamJ [6]. In the absence of *mamJ*, MamK filaments are no longer associated with magnetosomes, and magnetosomes form an aggregate in MSR-1 [28]. MamJ’s magnetosome localization requires MmsF or MmxF, two distant Tic20 protein transporter homologs [20]. Further studies are required to identify how MmsF and MmxF recognize MamJ and other client proteins as a cargo for magnetosome targeting.

A particular challenge for species like MSR-1 and AMB-1 is positioning a straight chain in their spirillum-shaped cell, which has no obvious linear landmarks. This task is achieved by relying on the distinct cell membrane curvatures of these species: because of their helical cell shape, AMB-1 and MSR-1 have areas of positive curvature, where the inner cell membrane is convex [22]. Similar to a fireman’s pole running down the center of a spiral staircase, the magnetosomes follow the positive curvature of the cell body to maintain a linear chain. MamY is a membrane protein that localizes to the positive curvature and, like MamK, relies on MamJ to connect to magnetosomes. MamY recognition of the positive cell curvature is driven by self-interaction and appears to be an intrinsic property of the protein, since it localizes similarly in a heterologous host [22]. In the absence of *mamY*, the magnetosome chain is localized to the incorrect curvature, and the cell is less efficient at orientating with a magnetic field [22,29]. Interestingly, MamK and MamY appear to act as two independent systems where the former pushes magnetosomes into a single chain and the latter localizes the chain to the positive curvature.

Although MSR-1 and AMB-1 are closely related MTB, there are a number of differences in their magnetosome chain assembly. For example, while MSR-1 has only one copy of *mamK*, AMB-1 has a second copy dubbed *mamK-like* [30]. MamK-like interacts with MamK, contributes to magnetosome chain formation, and increases MamK filament turnover [31]. Similarly, AMB-1 has multiple homologs of MamJ, two of which–MamJ and LimJ–appear to play redundant roles [27,29]. Perhaps the most visible difference between MSR-1 and AMB-1 is the organization of the magnetosome chain. In MSR-1, older magnetosomes are found at the chain center, and newer magnetosomes, many of which have not initiated biomineralization, are at the chain’s edge (Figure 2b). In contrast, AMB-1’s magnetosome chain features subchains of either older or newer magnetosomes that join end-to-end (Figure 2c) [29]. The distinct AMB-1 organization is produced by the action of McaA and McaB. McaA localizes to the positive cell curvature, and McaB localizes to magnetosomes that have started biomineralization. Analysis of *mcaA* and *mcaB* deletions demonstrates that the differences in chain organization are correlated with alterations in the directionality of MamK dynamics. One model suggests that McaA and McaB direct MamK to group older, crystal-containing magnetosomes into shorter subchains by promoting more local turnover of MamK filaments. This allows new magnetosomes to form in the space between these subchains. Thus, modulating MamK dynamics leads to a change in global organelle organization [29].

The mechanisms of magnetosome positioning described so far are based on work done on *Alphaproteobacteria* MTB. However, MTB are diverse and include species throughout the *Proteobacteria*, *Nitrospirae*, and several other phyla [32,33], many of which have complex chain architectures. For example, *Magnetobacterium bavaricum* fashions its hundreds of magnetosomes into an elaborate rosette-like arrangement organized around tube-like filamentous structures [28]. Fortunately, *Desulfovibrio magneticus* RS-1 (RS-1 for simplicity) has emerged as a *Deltaproteobacteria* model to reveal conserved and divergent aspects of magnetosome cell biology. RS-1 has a linear magnetosome chain localized to the positive cell curvature [34], but unlike AMB-1 and MSR-1 magnetosomes, which are lipid-bound [23,35], no membranes have been observed surrounding RS-1 magnetosomes [2]. Recent work shows that RS-1 uses a unique combination of actin-like proteins and coiled-coil proteins to position its membrane-less organelles [36]. In place of a lipid membrane, it seems possible that Mad10, a coiled-coil protein that has been described to bind magnetite, creates a proteinaceous casing around the magnetosomes on which other positioning proteins can bind and act upon [36,37]. All coiled-coil proteins involved in RS-1 magnetosome positioning are from a set of magnetosome proteins, named the Mad proteins, that are unique to *Deltaproteobacteria* and other phylogenetically distant MTB [32,36]. Thus, it is hypothesized that the coiled-coil protein system evolved as a distinct magnetosome positioning system in deep-branching MTB [36]. Exploring the diversity of MTB will expand our knowledge of both the MamK-MamJ-MamY system and novel magnetosome positioning mechanisms.

### Polyhydroxybutyrate and polyphosphate granules — tethered to the nucleoid

When growing in an abundance of carbon, some bacterial species will store the excess carbon in the form of a PHB granule [38]. PHB granules, also known as carbonosomes [39], are composed of a hydrophobic PHB core surrounded by a layer of proteins that synthesize, depolymerize, and regulate the number, size, and localization of the granule [38]. *pha* genes required for forming PHB granules are found in a wide range of bacteria and archaea, highlighting their potentially broad relevance to cellular health and survival [40,41]. PHB granules have also attracted wide ranging interest in applied settings since the carbon stored within them is a bioplastic [38].

**Figure 3:**
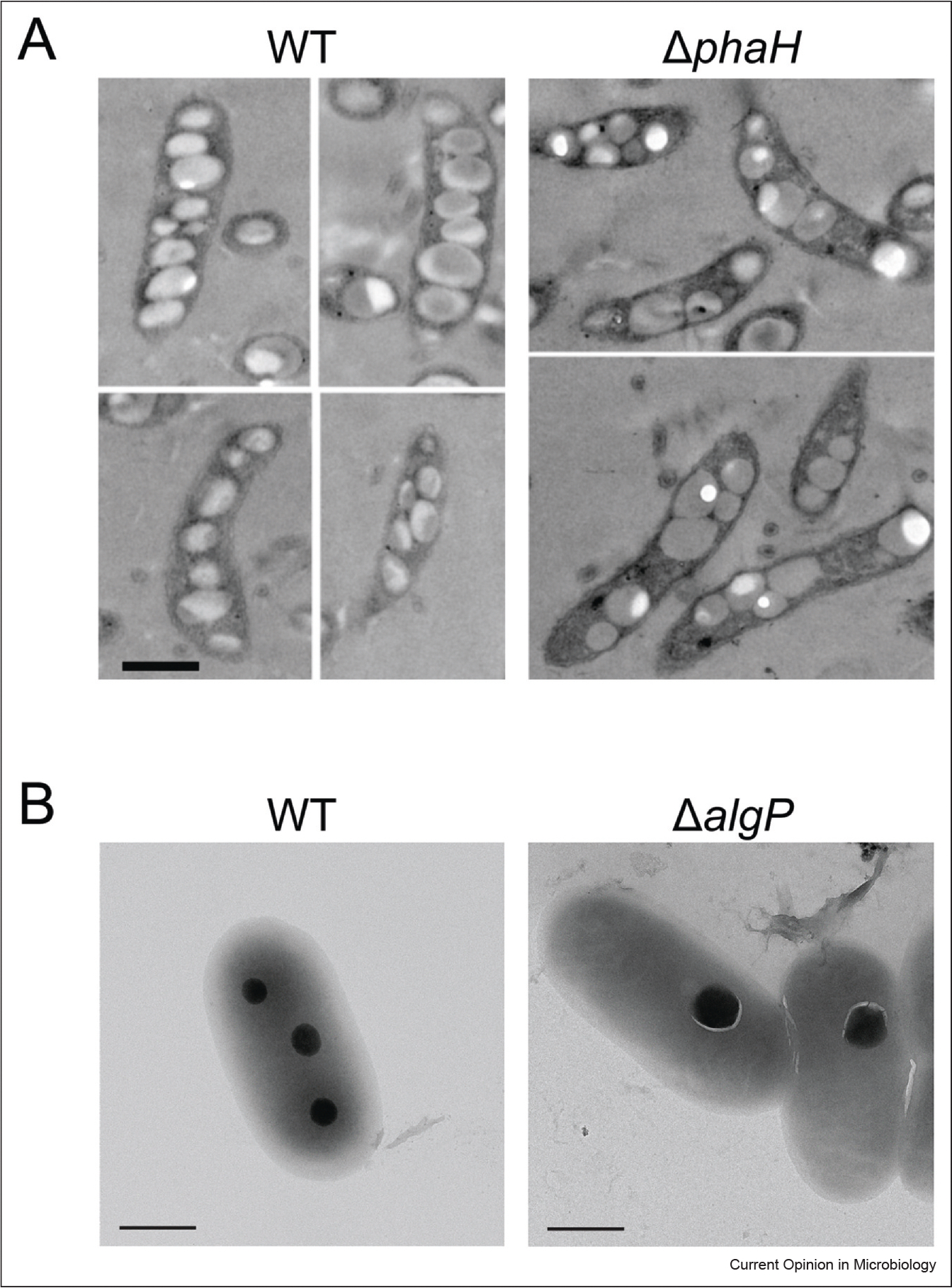
PHB and polyP granules are positioned by DNA-binding proteins. **(a)** Electron microscopy images of WT *C. crescentus* show PHB granules linearly positoned along the cell length. When *phaH* is deleted, the PHB granules are disorganized and have more aggregations [47]. Scale bar represents 1 μm. **(b)** Transmission electron microscopy of WT *P. aeruginosa* shows several, equally-spaced polyP granules. In contrast, the Δ*algP* strain has fewer, enlarged polyP granules [53]. Scale bar represents 0.5 μm. **(a)** Reprinted from ref. [47]. **(b)** Reprinted from ref. [53].

Though the arrangement can vary from species to species, PHB granules are typically positioned along the cell length to prevent aggregation and ensure equal segregation [42–45]. PHB granules are also large compartments that can take up a substantial volume of the cell, thus proper PHB granule placement might be necessary to reduce interruptions to other cellular processes [46]. *Cupriavidus necator* (also known as *Ralstonia eutropha*), *Pseudomonas putida*, and *Caulobacter crescentus* equally distribute their PHB granules along the nucleoid, but use different proteins to achieve that positioning: PhaM, PhaF, and PhaH, respectively (Figure 3a) [5,43,46,47]. All three proteins use their N-terminal end to bind to PHB granules and their C-terminal end to bind DNA nonspecifically [5,43,47].

Although the nonspecific DNA binding aspect is shared between PHB granule and carboxysome positioning, the three Pha proteins have no homology to ParA. PhaM and PhaF use a histone-like domain to bind DNA, while PhaH relies on a helix-hairpin-helix domain [5,43,47]. Random positioning through nonspecific attachment to the nucleoid could be sufficient to prevent aggregation of PHB granules, however it is also possible that additional undiscovered elements are involved. For example, perhaps the localization of the PhaM, PhaF, and PhaH is influenced by another protein, and the spacing between granules is achieved through a system analogous to that of carboxysome positioning. Another alternative mechanism is that PHB granules are actively repelled from one another to regulate the distance between them.

In *C. necator*, PHB granule position is closely tied with granule number and size. While WT *C. necator* has up to six PHB granules, *C. necator* Δ*phaM* instead has one or two oversized PHB granules [5,42]. Furthermore, when *phaM* is overexpressed, cells contain many smaller PHB granules that aggregate [5,42]. PhaM is known to activate the PHB-synthesizing enzyme PhaC, therefore the increased number of PHB granules in *phaM* over-expression strains is likely due to elevated PhaC activity [48,49]. PhaM complexes with PhaC, and together they are recruited to PHB granules during the early stages of granule formation and could be restricting the granule localization thereafter [45]. *C. necator* PhaM is sufficient to suppress polar localization of heterologously produced PHB granules in *Escherichia coli* [50]. Despite these advances, the biochemical mode of PhaM action in PHB granule organization remains unclear.

In *Pseudomonas putida*, a *phaF* deletion also results in aggregated PHB granules, although granule size does not change [43]. PhaF contribution to PHB granule position could be related to its interaction with PHB granule-associated PhaI or transcription regulator PhaD [51,52]. The DNA-binding domain of PhaF shares a high degree of homology with that of another Pseudomonad protein AlgP [53]. Interestingly, AlgP is responsible for the equal spatial distribution of a second intracellular structure, the polyphosphate (polyP) granule (Figure 3b) [53]. Removing the DNA-binding C-terminal domain of AlgP or deleting its gene completely in *Pseudomonas aeruginosa* causes the formation of fewer, enlarged polyP granules [53,54]. This phenotype is speculated to arise from increased polyP granule fusion that occurs when granule position is no longer regulated [53]. The parallels of PhaF and AlgP demonstrate a possible case where a strategy is recycled to position different compartments.

Given the large number of bacterial PHB and polyP granule producers, there is variation in how these compartments are organized across species. For example, in *C. crescentus*, polyP positioning is reliant on the cell cycle and chromosome segregation [55]. In early stages of cellular growth, a single polyP granule is found at the midcell. Before cell division, a second polyP granule forms, and the two granules are repositioned to the future middle of each daughter cell. This remarkable polyP granule localization is lost when chromosome replication or segregation is disrupted, suggesting that chromosome segregation is driving the granule repositioning. The dependence on proper chromosome segregation strongly implies that polyP granules in *C. crescentus* also require association with the nucleoid, but this is not yet confirmed, and the proteins responsible for this positioning have not been identified [55]. Other polyP granule organizations include polar localization, as in *Agrobacterium tumefaciens* and *Corynebacterium glutamicum* [56,57]. Further investigation of PHB and polyP granules can reveal the diverse evolutionary strategies used to position functionally and structurally similar compartments in species with different morphologies or physiologies.

## Conclusion

Examination of the examples above reveals a trend of the typical components found within bacterial organelle positioning systems (summarized in Table 1). In all cases, an element of the cell, such as the nucleoid or the cell membrane, serves as the scaffold to guide organelle organization. The scaffold is marked by a specific protein that works in concert with a second component, an organelle-bound protein. In some cases, a single protein performs both functions, as seen with the Pha proteins and AlgP [5,43,47,53]. A third component determines the spacing between organelles. For instance, McdA’s dynamic distribution across the nucleoid results in evenly spaced carboxysomes [8]. On the other hand, magnetosomes are pushed together to center the chain by MamK [25]. The nonspecific DNA-binding activity of PhaM, PhaF, PhaH, and AlgP may be sufficient to generate the uniform distribution of PHB and polyP granules, or there may be additional proteins involved that have not been uncovered yet [42,43,47,53]. Interestingly, MamK and McdA use ATP hydrolysis to drive organelle positioning [8,24]. It remains to be seen if similar expenditure of energy is needed for PHB and polyP granule localization.

Given the diversity of mechanisms discussed here, it is almost certain that there are strategies of bacterial organelle positioning left to be discovered. For example, encapsulins are well-described in their predicted function and applicative utility, but their cell biology is underexplored [3]. Innovative methods are essential for establishing and expanding on organelle organization model systems. One such experimental technique is the use of an unbiased, imaged-based screen to find changes in protein or organelle localization. These microscopy screens take advantage of high-throughput fluorescence imaging, which generally involves imaging strains in a multi-well format with automated well-to-well stage movement and auto-focusing software [58–60]. Additionally, bacterial organelle research can be further enhanced by increasing accessibility to high-resolution microscopy techniques, such as cryo-electron tomography and super-resolution fluorescence microscopy, allowing cell biologists to overcome the miniscule size of bacterial compartments [3]. An upcoming solution is the development of expansion microscopy, in which an organism is isotropically enlarged in a hydrogel to increase spatial resolution [61]. Though methods are still being adapted for bacterial samples [62,63], maturation of this technique and others will expand the boundaries of bacterial cell biology.

Defining pathways of organelle assembly and positioning can also be critical in engineering and scaling up production of organelles in applied settings. For example, there are currently efforts to heterologously express carboxysomes in both bacterial and eukaryotic hosts for purposes such as optimizing photosynthesis or redesigning the carboxysome to hold novel cargo [64]. Knowledge about carboxysome positioning and segregation is vital for ensuring that these structures are retained in the hosts for many cell divisions. In another case, the magnetic properties of magnetosomes have formed the basis for targeted drug delivery through payload-attached MTB that are guided toward specific tissue with a magnetic field [65]. Alignment and response of MTB cells to the magnetic field–driven by magnetosome organization–is a critical component for the success of these applications. Indeed, the cell biology of bacterial organelles cannot be ignored in many applicative contexts. Thus, the study of organelle positioning not only provides a context to examine broader aspects of bacterial cell biology, but will also make biotechnological advances more attainable. With increasing efforts and improving technology, we look forward to future discoveries in bacterial organelle organization.

## Supplementary Material

Supplemental Materials

## Figures and Tables

**Table 1:** Summary of scaffold binding, organelle binding, and organelle spacing components of bacterial organelle positioning systems reviewed here.

Organelle	Organism (s)	Scaffold	Scaffold binding	Organelle binding	Organelle spacing	ATP-dependent?	Ref.

Carboxysome	*S. elongatus* PCC 7942, *H. neapolitanus*	Nucleoid	McdA	McdB	McdA	Yes	[8,9]
Magnetosome	*M. gryphiswaldense* MSR-1, *M. magneticum* AMB-1	Cell Membrane	MamY	MamJ	MamK	Yes	[6,22,25]
PHB Granule	*C. necator*, (*R. eutropha* H16)	Nucleoid	PhaM	PhaM	PhaM/?	Unknown	[5,42,45]
	*P. putida*		PhaF	PhaF	PhaF/?		[43,51]
	*C. crescentus*		PhaH	PhaH	PhaH/?		[47]
PolyP Granule	*P. aeruginosa*	Nucleoid	AlgP	AlgP	AlgP/?	Unknown	[53]

## Data Availability

No data were used for the research described in the article.

## Appendix A. Supporting information

Supplementary data associated with this article can be found in the online version at doi:10.1016/j.mib.2025.102614.
